# KL-6 in ARDS and COVID-19 Patients

**DOI:** 10.37825/2239-9754.1035

**Published:** 2022-09-16

**Authors:** Ornella Piazza, Giuliana Scarpati, Giovanni Boccia, Massimo Boffardi, Pasquale Pagliano

**Affiliations:** a Department of Medicine, Surgery and Dentistry “Scuola Medica Salernitana”, University of Salerno, Via Salvatore Allende, 84081, Baronissi Salerno, Italy; b AOU San Giovanni di Dio e Ruggi d’Aragona, P.O. Cava de’ Tirreni (SA), Salerno, Italy

**Keywords:** KL-6, MUC1, ARDS, COVID-19

## Abstract

The Acute Respiratory Distress Syndrome (ARDS) is a common, devastating clinical pattern characterized by life-threatening respiratory failure. In ARDS there is an uncontrolled inflammatory response that results in alveolar damage, with the exudation of protein-rich pulmonary-edema fluid in the alveolar space. Although severe COVID-19 lung failure (CARDS) often meets diagnostic criteria of traditional ARDS, additional features have been reported, such as delayed onset, binary pulmonary compliant states, and hypercoagulable profile. Increased levels of Krebs von den Lungen 6 (KL-6, also known as MUC1) have been reported in both ARDS and CARDS. KL-6 is a transmembrane protein expressed on the apical membrane of most mucosal epithelial cells and it plays a critical role in lining the airway lumen. Abnormalities in mucus production contribute to severe pulmonary complications and death from respiratory failure in patients with diseases such as cystic fibrosis, chronic obstructive pulmonary disease, and acute lung injury due to viral pathogens. Nevertheless, it is not clear what role KL-6 plays in ARDS/CARDS pathophysiology. KL-6 may exert anti-inflammatory effects through the intracellular segment, as proven in animal models of ARDS, while its extracellular segment will enter the blood circulation through the alveolar space when the alveolar epithelial cells are damaged. Therefore, changes in plasma KL-6 levels may be useful in ARDS and CARDS phenotyping, and KL-6 might guide future clinical trials in ‘personalized medicine’ settings.

## 1. Introduction

In acute respiratory distress syndrome (ARDS) there is an uncontrolled inflammatory response that results in alveolar damage, with the exudation of protein-rich pulmonary-edema fluid in the alveolar space, eventually causing respiratory failure [1]. ARDS is characterized by an acute onset of lung injury, which can be caused by a variety of pulmonary or extra-pulmonary diseases. Subsequent development of non-cardiac lung edema causes hypoxemia, which may lead to multi-organ failure and death. The more common precipitating conditions are: pneumonia (pulmonary ARDS), aspiration of gastric contents, inhalational injury, contusion, vasculitis and near drowning. Among the indirect risk factors there are: sepsis from an extra-pulmonary source, non-cardiogenic shock, severe trauma, pancreatitis, extensive burns, drug overdose and multiple blood transfusions. Pathologically, ARDS is characterized by diffuse alveolar damage (DAD), and evolves over 2 or 3 weeks through exudative, inflammatory and fibro-proliferative phases. Superimposed cardiac failure, pneumonia, pulmonary embolism, ventilator-induced lung injury, malposition of tubes, central venous catheters and drainages or other conditions may suddenly worsen the clinical evolution. Progressive lung fibrosis and pulmonary hypertension can be infrequent complications of ARDS. From its first description in 1967, a number of prospective studies have demonstrated that ARDS is not rare, as its frequency is reported to be between 14 and 86 persons per 100,000 per year in a general adult population [2,3], but there is a great variability when focused intervention are set up as in Olmsted County, where incidence descended from 81 to 38.3 cases per 100,000 person-years driven by reduction in ‘hospital acquired’ ARDS [4].

Although severe coronavirus disease 2019 (COVID-19) lung failure often meets diagnostic criteria of traditional ARDS, additional features have been reported, such as delayed onset, binary pulmonary compliant states, and hypercoagulable profile. The efficacy of steroids in COVID-19 [5–7] and need for systemic anticoagulation have been established. Despite its novelty, COVID-19 ARDS (CARDS) has clear crossover with traditional ARDS therapy, and lung-protective ventilation and prone positioning should be widely used [8].

Mortality among adults with ARDS has been reported as 24%–60%, depending on age and underlying health status of the patient. Hospital mortality increases with ARDS severity and hypoxia, which is the landmark of this syndrome [2–4]. The severe hypoxia that characterizes ARDS is mainly due to a ventilation-perfusion mismatch, resulting in increased intrapulmonary shunting due to pulmonary vasodilatation in non-ventilated lung regions and vasoconstriction in ventilated areas, as well as pulmonary hypertension. Diagnosis of ARDS is mainly clinical, but corroborated by Chest X-Ray or CT scan, showing a severe lung edema, which is not fully explained by cardiac failure or fluid overload, and bilateral opacities not entirely explicated by effusions, lobar/lung collapse or nodules. The typical computed tomography features of ARDS show: non-homogeneous distribution, a ventrodorsal gradient of density, more dense consolidation in the dependent regions, widespread ground-glass opacities associated with thickening of interlobular septa (crazy paving), and pleural effusion. Signs and symptoms are not specific (mainly dyspnoea, cyanosis, tachypnea and hypoxemia) and mimic those of pulmonary edema. In CARDS clinical manifestations can be not very prominent in many hypoxic patients, with no complaint of dyspnea, no significant increase in respiratory rate, and no respiratory distress (the so called “silent hypoxia”) [9]. Therefore, the need for specific biomarkers for ARDS exists, to enhance prediction of ARDS development, allowing early targeted interventions or helping to identify ‘at risk’ patients, in whom physician should apply a ‘bundle’ of strategies to prevent ARDS, including optimal mechanical ventilation, aggressive resuscitation, reduction in transfusion, prevention of common complications. Starting from biology of ARDS, we can distinguish biomarkers of lung epithelial injury, inflammatory response, lung endothelial injury. Biomarkers may help to distinguish ARDS caused by direct lung injury or by indirect lung injury. Direct lung injury is characterized by a molecular phenotype consistent with more severe lung epithelial injury and less severe endothelial injury. The opposite pattern was identified in indirect lung injury. ARDS caused by direct lung injury has significantly higher levels of surfactant protein-D, a biomarker of lung epithelial injury but significantly lower levels of a biomarker of endothelial injury (angiopoietin-2). Among the lung epithelium damage biomarkers, there is Krebs von den Lungen 6 (KL-6) also known as mucin 1 (MUC1) [10].

### 1.1. KL-6 as a biomarker to phenotype ARDS and CARDS

KL-6 is a transmembrane protein expressed on the apical membrane of most mucosal epithelial cells, which plays a main role in lining the airway lumen [11]. In healthy individuals, mucus along the lumen serves as a major protective barrier against inhaled pathogens, toxins, and other foreign particles. However, excessive mucus in the airways has been linked to increased frequency and duration of infections, decreased lung function, and increased mortality from respiratory diseases. Abnormalities in mucus production contribute to severe pulmonary complications and death from respiratory failure in patients with diseases such as cystic fibrosis, chronic obstructive pulmonary disease (COPD), and acute lung injury due to viral pathogens, such as SARS-CoV2. Overexpression, aberrant intracellular localization, and changes in glycosylation of this protein have been associated with carcinomas. In the cell nucleus, KL-6 regulates the activity of transcription factor complexes that have a documented role in tumor-induced changes of host immunity. Type II pneumocytes damage or transformation is a very relevant element in both CARDS and ARDS by other viral causes since, in case of alveolar type II cells injury, removal of alveolar edema fluid is compromised. Moreover, harm to type II cells reduces the production and turnover of surfactant, which is associated with poor outcome. In a sepsis study [12], plasma KL-6 levels of septic patients with ARDS were compared to a group of septic patients without ARDS. KL-6 levels higher than 1.37 ng/ml on the day of admission or higher than 2.35 ng/ml on the third day identified patients with sepsis with the highest risk of ARDS. KL-6 levels in the survivors group were lower than that in the non-survival group, both on the day of enrollment and on the 3rd day.

Frix [13] showed that serum KL-6 levels in COVID-19 patients were increased compared to healthy subjects, but to a lesser extent than in patients suffering from interstitial lung disease (ILD). Increased levels of KL-6 in COVID-19 patients were associated with a more severe lung disease. Moreover, D'Alessandro et al. [14] demonstrated that KL-6 serum concentrations were significantly higher in COVID-19 severe patients than the in non-severe patients (P = .0118), with a cut-off value of 406.5 U/ ml. In our experience ([15], and in press data) at Salerno University COVID ICU, on 122 very severe patients, at ICU admission, KL-6 serum level was significantly lower in the survivors group (median value 545 U/ml vs 1070 U/ml in those patients who did not survive at 28-day after ICU admission) and in patients that were managed with Non-Invasive Ventilation (NIV) for the whole length of ICU stay (711 U/ml vs 1073 U/ml in patients who required endotracheal intubation at the admission or during their ICU stay). KL-6 was not correlated with gender.

Nevertheless, many attempts are still ongoing to profile COVID patients with proper biomarkers and different approaches [16].

## 2. From KL6 increase in serum to a possible role as drug target

It is not yet clear which is the role of KL-6 in ARDS. Ye et al. [17] investigated the effect and mechanism of the Mucin 1 (MUC1) gene and its recombinant protein on lipopolysaccharide (LPS)-induced ALI/ARDS. Overexpression of MUC1 effectively ameliorated LPS-induced damage to BEAS-2B cells and, in an animal model, LPS successfully induced ALI/ARDS in mice, while MUC1 attenuated lung injury. MUC1 also reduced the expression of inflammatory factors (IL-1β, TNF-α, IL-6 and IL-8) and oxidative stress levels in mice. MUC1/KL-6 mainly exerts anti-inflammatory effects through the intracellular segment during the occurrence of acute lung injury caused by sepsis. Its extracellular segment will enter the blood circulation through the alveolar space when the alveolar epithelial cells are damaged so changes in plasma MUC1/KL-6 levels can effectively reflect lung injury. Even if the role of serum KL-6 in ARDS and in COVID-19 is still uncertain, Kost-Alimova et al. [18] proposed R406, the active metabolite of Fostamatinib, as a compound for the reduction of KL-6 levels in lung epithelium in the setting of COVID ARDS. Fostamatinib is a tyrosine kinase inhibitor medication for the treatment of chronic immune thrombocytopenia. R406 is a potent inhibitor of spleen tyrosine kinase (SYK), a cytosolic protein tyrosine kinase required for the expression of several pro-inflammatory cytokines. R406 (at EC50 concentration) substantially reduced MUC1 abundance in or near the plasma membrane, with a portion of MUC1 retained in cytosolic and perinuclear cell compartments. Fostamatinib, a Food and Drug Administration (FDA)-approved drug, has been proposed also to stop neutrophils release of neutrophil extracellular traps (NETs) during systemic bacterial or viral infection. A trial is ongoing (ClinicalTrials.gov Identifier: NCT04579393).

## 3. Conclusion

Krebs von den Lungen 6 is a mucin-like glycoprotein expressed on the surface membrane of alveolar epithelial cells and bronchiolar epithelial cells. KL-6 serum levels could be useful for detecting the presence of alveolar epithelial cells injury, evaluating ARDS activity, response to therapy, predicting clinical outcomes.

However, no single ARDS/CARDS biomarker is likely to meet all needs but combining panels of biomarkers with different sources shows promise. Moreover, combining biomarkers with clinical data enhances predictive validity. KL-6 may play a key role in ARDS phenotyping, to guide future clinical trials in ‘personalized medicine’ settings.
